# How Shall the Stomatologist Be Educated?*Read in the Section on Stomatology of the American Medical Association at the Sixty-first Annual Session, at St. Louis, June, 1910.

**Published:** 1910-07-15

**Authors:** Eugene S. Talbot

**Affiliations:** Chicago


### HOW SHALL THE STOMATOLOGIST BE EDUCATED?*

EUGENE S. TALBOT, M.D., D.D.S., CHICAGO.

It is a popular idea that "the dentist on graduation is better equipped for practice than the physician." This is misleading, not only to the dental graduate, but also to the physician and the laity. The statement was tenable seventy years ago when the first dental college was established and dental mechanics only were practiced, but modern research has shown us that many incipient diseases first show themselves in the mouth by their effect on the mucous membrane, alveolar process, jaws and teeth. These conditions frequently become serious.

In conversation with some of the members of the Council on Medical Education, in regard to the medical education of the dentist, they were surprised to know that further education was necessary. They were under the impression that the graduate of dentistry had received a full and complete education; that is, all that was necessary to fit him for his special branch of medicine.

There are a few dentists who, after they have been in practice a short time, realize that they know little of the treatment of diseases of the mouth, so necessary at the present time. Some are willing and able to take a medical course to prepare themselves thoroughly to become stomatologists. Others would like to do so but circumstances will not permit it.

### NO CREDIT FROM THE MEDICAL SCHOOLS

The medical school does not give credit for any part of the course of the dental graduate (and rightfully), because he has scarcely a smattering of the fundamentals of medicine.

---

*Read in the Section on Stomatology of the American Medical Association at the Sixty-first Annual Session, at St. Louis, June, 1910.

It is necessary for the dentist to take a medical course of four years, over and above the three years spent in the dental school. In the best dental schools, the Dean will tell you the dental students sit in the same room, hear the same lectures, and stand the same examinations as the medical students All know the dental student is not required to have the close marking required of the medical. What a disgrace for the dental graduate after a three years' course in one department of a university to have so vague a knowledge of the fundamentals of medicine that he cannot get one year's credit in another department in the same universty although he has been sitting on the same seat, in the same room and hearing the same teacher on the same subject. Teachers on the fundamentals of medicine have informed me many times that dental students did not need the same serious consideration of the subject as the medical students required, and therefore their examination was not so severe; hence, the dental student is allowed to slip through on a lowe average than the medical student.

The dental student on graduation knows little or nothing of the fundamentals of medicine, no matter how well he may have been taught. The reason is that the dental student on matriculation, is immediately put to work on the mechanics of his profession. The entire environment and atmosphere of the school is mechanics. The contrast between the mechanic and the biologic is so great that the student immediately asks the question. "What is the advantage of all of this study of anatomy, physiology, pathology, chemistry and therapeutics when the care of the teeth is all I require?" Thus the atmosphere of the school itself makes the student a mechanic rather than a broadly educated man.

### THE DEGREE OF D.D.S.

Some thinkers are trying, or rather expecting to raise the standard of the D. D. S. to equal the M. D. degree, and thus to an honorable position. To my mind, it can never be accomplished. The D.D.S. will always be considered an inferior degree. To make the degree equal to the medical the student must study the same number of years. Then why not confer the medical degree? The student will not tolerate an inferior degree. Present methods of dental teaching will not improve the student's mentality though a six years course be required for graduation.

### STOMATOLOGY AS A SPECIALTY.

There can be no question that men proficient in the mechanics of dentistry (to repair and restore lost parts), and men to treat diseases of the mouth (which includes the jaws and alveolar precesses), are essential. Shall the specialty be divided and the dentist confine himself solely to the mechanics of the teeth and the stomatologist to the diseases of the mouth, or shall the student be so educated as to combine both?

If the specialty is to be divided, dental schools are wasting time and money in even attempting to teach the fundamentals of medicine. There are, in the United States, fifty-four well-equipped schools to teach the mechanics of dentistry, and they are all that can be desired in this direction. The stomatologist, on the other hand, is losing time in not acquiring a better foundation for the practice of his specialty. He should matriculate in the medical school and obtain a thorou h medical education, since modern research has demonstrated that not a single disease of the mouth, jaws and teeth but has a systematic relationship. Personally, I am of the opinion that the two departments, pertaining to all operations in the mouth, cannot readily be separated. The question is then, "How Shall the Future Stomatologist be Educated?"

### INTERNATIONAL ASSOCIATION OF STOMATOLOGY.

In August, 1907, there was organized in Paris, France, The International Association of Stomatology. The delegates to that meeting all held medical degrees from universities and colleges in the various countries from which they came. Nearly every country was represented. The object of this organization is to urge a medical education alike in all countries for those practicing diseases of the oral cavity.

At this meeting, two questions were settled: First, That a complete medical education must be required of every dentist. Second, the word "stomatology" was defined as meaning the science that treats of diseases pertaining to the mouth; the "stomatologist" is one who treats diseases of the mouth.

THE QUESTIONS TO BE SETTLED.

At the second meeting of the A. S. I., held at Budapest, August 1909, the following paper by me, "How Shall the Stomatologist be Educated?" was discussed. The thoughts therein expressed were incorporated in a report prepared for universal discussion. These questions are to be sent to members in the various countries for careful study, and are here presented for your consideration. They are as follows:

*a.* When is it best to begin the special studies; during the time of the general medical studies or immediately after these'

*b* Advantages and disadvantages of each.

1.Special studies begun during the special medical course.

2. Special studies begun immediately after the general medical course.

*c.* Length of time required for special studies of each system.

*d.* Details of special studies of each system.

It is necessary that these questions be seriously considered by the members of this section, since they must serve as the foundation for future dental education in many, if not all, the countries, and also for the lengthy discussion before the A. S. I. at its next meeting in Paris, 1911. It will be necessary also to publish this discussion in full in the *Bulletin* for 1910, Volume III, from the *Journal* of the American Medical Association, in order that it may appear some months previous to that meeting, so that an intelligent understanding of the subject may be presented from each country.

The fathers of American medicine, the late Drs. N. S. Davis, Gross, Sayre, Flint, Toner, Pancost, and many others, believed that diseases of the mouth, including oral hygiene, the most important study of the human body. Stomatologists are of the same opinion. This being the case, should not this specialty require the same method and the same length of study as the lesser specialties of medicine? The dental profession has not yet awakened to the seriousness, importance and scope of its calling.

The main point and first to be considered is, how can the stomatologist be educated and not lose time, but have all his studies count for future work. At the present time, ther are two methods of studying medicine in the United States, hence the education of the stomatologist must be based on these methods, subject to more modern improvements. Both medical and dental students, at the present time, must possess a high school diploma or its equivalent; therefore, they start their chosen profession on an equal footing. In some medical schools, the high school graduate is allowed to matriculate, pursue a four years' course and graduate with an M.D. degree. The other method hasbeen worked out by Dr. John Milton Dodson[1] as follows:

"With the last decade there has developed a plan in several American universities under which a student takes, during the junior and senior years, those sciences which constitute the first two years of the medical curriculum. Credit for the successful completion of the work in these branches is permitted to count both toward a baccalaureate

---

1. THE JOURNAL A. M. A., Sept. 6, 1902, and May 22, 1909.

degree in the collegiate department and toward the degree of M.D. in the medical school. The student is thus able to secure the baccalaureate degree and the degree of M.D. in from six to seven years after his completion of th ehigh school course and his matriculation in the university. The plan has extended rapidly, it being now in operation in over thirty American universities. It is believed by many deucators to be the best solution yet offered of the problem of how to provide a good many students with a fairly adequate premedical training, and to induct them into the study, and later, the practice of medicine at a reasonably early age."

This method of teaching will, no doubt, become universal in this country. By either method the dental student must pursue the fundamentals of medicine in physics, zoology, chemistry, anatomy, physiology, embryology, bacteriology, pharmacology and the principles of pathology.

The first two years of medical studies should be conducted in the universities proper since they are better equipped than the average medical college for this work. The stomatologist should continue the course to this point with the medical class and obtain credit. He should not come in contact with patients during the first two or three years of study Laboratory work and the use of the microscope has given him considerable experience in finger manipulation. The finger manipulation cannot be more delicate than that required in the laboratory and dissecting room of a university or medical school. To offset this objection, if it can be considered one, it is essential that the student should be required to obtain as much of his professional education as possible before coming in contact with the commercial side of his specialty, which is so demoralizing to his future welfare by the present methods of teaching.

One strong argument put forth against a medical education for the dentist is the early requirement of finger skill which is supposed to be lost later in life, the student being too old after a medical education to acquire the skill necessary to perform operations in the mouth.

To ascertain the ages of entrance into the best schools in the country, I wrote to eight, requesting them to send me a report of the ages of matriculation for the past five years. Deans Koch, of the Northwestern University Dental School; Smith, of Harvard University Dental School; Kirk, of the University of Pennsylvania Dental School, and Hoff, of the University of Michigan Dental School, responded.

TABLE 1.—AGES OF STUDENTS OF NORTHWESTERN UNIVERSITY DENTAL SCHOOL AT THE TIME OF ENTRANCE.

| Age | 1904 | 1905 | 1906 | 1907 | 1908 | 1909 | Total Students | Total Years |
|---|---|---|---|---|---|---|---|---|
| Eighteen | 13 | 24 | 8 | 6 | 4 | 6 | 61 | 1,098 |
| Nineteen | 19 | 23 | 8 | 8 | 11 | 13 | 82 | 1,558 |
| Twenty | 22 | 23 | 14 | 15 | 18 | 21 | 113 | 2,260 |
| Twenty-one | 22 | 34 | 6 | 19 | 14 | 27 | 122 | 2,562 |
| Twenty-two | 20 | 32 | 13 | 15 | 11 | 23 | 114 | 2,508 |
| Twenty-three | 21 | 24 | 13 | 8 | 6 | 17 | 89 | 2,047 |
| Twenty-four | 13 | 24 | 12 | 7 | 11 | 10 | 77 | 1,840 |
| Twenty-five | 15 | 12 | 6 | 12 | 4 | 11 | 60 | 1,508 |
| Twenty-six | 10 | 9 | 7 | 7 | 6 | 7 | 46 | 1,196 |
| Twenty-seven | 8 | 6 | 3 | 6 | 6 | 6 | 35 | 945 |
| Twenty-eight | 4 | 9 | 1 | 5 | 2 | 2 | 23 | 644 |
| Twenty-nine | 8 | 8 | 2 | 4 | 2 | 3 | 27 | 783 |
| Thirty | 2 | 3 | 3 | 3 | 7 | 4 | 22 | 660 |
| Thirty-one | 2 | 4 | 1 | 4 | 1 | 1 | 13 | 403 |
| Thirty-two | -- | 3 | 1 | 1 | 1 | 3 | 9 | 288 |
| Thirty-three | -- | 1 | -- | 4 | -- | -- | 5 | 165 |
| Thirty-four | 1 | 1 | -- | 3 | 2 | 1 | 8 | 272 |
| Thirty-five | 1 | 2 | -- | 1 | 1 | 1 | 6 | 210 |
| Thirty-six | 2 | 1 | 1 | 1 | 2 | -- | 7 | 252 |
| Thirty-seven | -- | 2 | -- | -- | 2 | 3 | 7 | 259 |
| Thirty-eight | 1 | 1 | -- | -- | -- | 1 | 3 | 114 |
| Thirty-nine | -- | 2 | -- | -- | -- | -- | 2 | 78 |
| Forty and over | -- | -- | 2 | -- | -- | -- | 2 | 80 |
| Total | 184 | 248 | 101 | 129 | 111 | 160 | 933 | 21,730 |

Average 23.29.

TABLE 2—AGES OF STUDENTS OF HARVARD UNIVERSITY DENTAL SCHOOL AT THE TIME OF ENTRANCE

| Age | 1905 | 1906 | 1907 | 1908 | 1909 | Total Students | Total Years |
|---|---|---|---|---|---|---|---|
| Sixteen | -- | 1 | -- | -- | -- | 1 | 16 |
| Seventeen | 1 | 4 | -- | 1 | 1 | 7 | 119 |
| Eighteen | 3 |  | 9 | 2 | 6 | 22 | 396 |

TABLE 2—Continued.—AGES OF STUDENTS OF HARVARD UNIVERSITY DENTAL SCHOOL AT THE TIME OF ENTRANCE.

| Age | 1905 | 1906 | 1907 | 1908 | 1909 | Total Students | Total Years |
|---|---|---|---|---|---|---|---|
| Nineteen | 7 | 6 | 5 | 9 | 14 | 41 | 779 |
| Twenty | 9 | 9 | 13 | 11 | 15 | 57 | 1,140 |
| Twenty-one | 12 | 11 | 7 | 11 | 15 | 56 | 1,176 |
| Twenty-two | 17 | 4 | 4 | 6 | 5 | 36 | 792 |
| Twenty-three | 8 | 7 | 4 | 3 | 7 | 29 | 667 |
| Twenty-four | 3 | 4 | 7 | 4 | 6 | 24 | 576 |
| Twenty-five | 2 | 2 | 2 | 4 | 2 | 12 | 300 |
| Twenty-six | 5 | 4 | 3 | 2 | 2 | 16 | 416 |
| Twenty-seven | 2 | 2 | 2 | 3 | 1 | 10 | 270 |
| Twenty-eight | 2 | 1 | 3 | 1 | 5 | 12 | 336 |
| Twenty-nine | 3 | 4 | -- | 3 | 2 | 12 | 348 |
| Thirty | 1 | 1 | 2 | 2 | 4 | 10 | 300 |
| Thirty-one | 4 | 1 | -- | 1 | 2 | 8 | 248 |
| Thirty-two | 2 | 1 | -- | 1 | 2 | 6 | 192 |
| Thirty-three | 1 | 1 | 2 | -- | -- | 4 | 132 |
| Thirty-four | -- | 1 | -- | 1 | -- | 2 | 68 |
| Thirty-six | 1 | -- | -- | -- | -- | 1 | 36 |
| Thirty-seven | -- | -- | -- | -- | 1 | 1 | 37 |
| Thirty-eight | 1 | -- | -- | -- | -- | 1 | 38 |
| Thirty-nine | 1 | -- | -- | -- | 1 | 2 | 78 |
| Forty | -- | 1 | 1 | 1 | -- | 3 | 120 |
| Forty-one | -- | -- | 1 | -- | -- | 1 | 41 |
| Forty-two | -- | -- | -- | -- | 1 | 1 | 42 |
| Forty-four | -- | -- | 3 | -- | -- | 3 | 132 |
| Total | 85 | 67 | 68 | 66 | 92 | 378 | 8,795 |

Average 23.29.

TABLE 3.—AGES OF STUDENTS OF UNIVERSITY OF PENNSYLVANIA DENTAL DEPARTMENT AT THE TIME OF ENTRANCE

| Age | 1905 | 1906 | 1907 | 1908 | 1909 | Total Students | Total Years |
|---|---|---|---|---|---|---|---|
| Seventeen | 3 | 3 | 3 | 2 | 2 | 13 | 221 |
| Eighteen | 13 | 17 | 22 | 16 | 21 | 89 | 1,602 |
| Nineteen | 25 | 40 | 40 | 26 | 26 | 157 | 2,983 |
| Twenty | 30 | 21 | 26 | 38 | 25 | 140 | 2,800 |
| Twenty-one | 31 | 33 | 29 | 23 | 28 | 144 | 3,024 |
| Twenty-two | 17 | 22 | 28 | 9 | 14 | 90 | 1,980 |
| Twenty-three | 11 | 13 | 23 | 12 | 7 | 66 | 1,518 |
| Twenty-four | 14 | 13 | 9 | 5 | 3 | 44 | 1,056 |
| Twenty-five | 9 | 8 | 6 | 5 | 4 | 32 | 800 |
| Twenty-six | 3 | 7 | 6 | 5 | 3 | 24 | 624 |
| Twenty-seven | 4 | 5 | 2 | 6 | 2 | 19 | 513 |
| Twenty-eight | 3 | 3 | 8 | 3 | 2 | 19 | 532 |
| Twenty-nine | 1 | 2 | 1 | 2 | 2 | 8 | 232 |
| Thirty | 2 | 1 | 1 | 1 | 1 | 6 | 180 |
| Thirty-one | 4 | 2 | 1 | -- | -- | 7 | 217 |
| Thirty-two | 2 | 1 | 1 | -- | -- | 4 | 128 |
| Thirty-three | -- | -- | -- | 1 | -- | 1 | 33 |

TABLE 3.—Continued.—AGES OF STUDENTS OF UNIVERSITY OF PENNSYVANIA DENTAL DEPARTMENT AT THE TIME OF ENTRANCE.

| Age | 1905 | 1906 | 1907 | 1908 | 1909 | Total Students | Total Years |
|---|---|---|---|---|---|---|---|
| Thirty-four | -- | -- | 1 | -- | 1 | 2 | 68 |
| Thirty-five | -- | -- | 1 | -- | -- | 1 | 35 |
| Thirty-six | -- | -- | 1 | -- | 1 | 2 | 72 |
| Thirty-eight | -- | -- | -- | -- | 1 | 1 | 38 |
| Forty | -- | -- | 1 | -- | -- | 1 | 40 |
| Forty-four | -- | -- | -- | -- | 1 | 1 | 44 |
| Total | 172 | 191 | 210 | 154 | 144 | 871 | 18,740 |

Average 21.53.

TABLE 4.—AGES OF STUDENTS OF THE UNIVERSITY OF MICHIGAN DENTAL SCHOOL AT THE TIME OF ENTRANCE.

| Age | 1905 | 1906 | 1907 | 1908 | 1909 | Total Students | Total Years |
|---|---|---|---|---|---|---|---|
| Eighteen | 4 | 1 | 5 | 2 | 3 | 15 | 270 |
| Nineteen | 3 | 4 | 11 | 8 | 10 | 36 | 684 |
| Twenty | 3 | 5 | 8 | 4 | 10 | 30 | 600 |
| Twenty-one | 7 | 1 | 8 | 9 | 9 | 34 | 714 |
| Twenty-two | 5 | 2 | 9 | 8 | 4 | 28 | 616 |
| Twenty-three | 1 | 2 | 3 | 4 | 9 | 19 | 437 |
| Twenty-four | 4 | 1 | 4 | 2 | 1 | 12 | 288 |
| Twenty-five | 5 | -- | 1 | 3 | -- | 9 | 225 |
| Twenty-six | 3 | 1 | -- | 3 | 1 | 8 | 208 |
| Twenty-seven | -- | -- | 1 | -- | -- | 1 | 27 |
| Twenty-eight | 1 | 2 | 4 | 1 | 2 | 10 | 280 |
| Twenty-nine | 1 | 1 | -- | 1 | -- | 3 | 87 |
| Thirty | -- | 1 | -- | 2 | 1 | 4 | 120 |
| Thirty-one | 2 | -- | -- | -- | 1 | 3 | 93 |
| Thirty-two | -- | -- | 1 | 1 | -- | 2 | 64 |
| Thirty-three | 1 | -- | -- | -- | -- | 1 | 33 |
| Thirty-four | -- | -- | 1 | -- | -- | 1 | 34 |
| Total | 40 | 21 | 56 | 48 | 51 | 216 | 4,780 |

Average 22.12.

### WHAT THE TABLES SHOW.

The reports are interesting since they show the youngest to be 16 and 17 and the oldest 44, with an average in Harvard and Northwestern of 23.29, and in the University of Pennsylvania 21.53. I have known students to matriculate in the dental school as late as 54 years of age. The ages of matriculation in all the other dental schools must range in similar manner. The fallacy of this argument must, therefore, fall to the ground.

In the university medical schools, the average is lower than in the regular medical schools. Thus in Harvard and Johns Hopkins the average is about twenty, while in the schools not connected with universities the average is from twenty-two to twenty-four. Students leave the high school in this country usually at eighteen to twenty years of age. The same is true of the gymnasium in Germany and the institutes of France:

The reason that medical and dental students matriculate late in life in schools not departments of universities is that most of these students have started out pursuing other avocations. Not making a success in their chosen calling or having a taste for the professions they have sought new fields for livelihood. Few of these men have taken a high school course or have even passed successfully through the grammar school. Every year the people are becoming more exacting in regard to the education of students for the professions. This will necessitate the students entering the profession direct from the high school, thus reducing the age of matriculation to a minimum. Students of dentistry entering the medical school at twenty can graduate with both medical and dental degrees at twenty-six, which is early enough for any man to begin the practice of a profession.

The stomatologist, having finished his two or three years of the fundamentals of medicine, now has the choice of two methods. He can finish his full course in medicine before taking up his specialty, or he can take three months in the summer for his clinical work and finish in one or two extra years, thus cutting off one year in his special course and adding one to his medical course. In either case he has spent only six years as against the seven or eight for other specialties. This is certainly not too much to expect of a department of medicine recognized by medical practitioners as the most important specialty.

### THE ELECTIVE SYSTEM.

The elective system in medicine, as recommended by ex-President Eliot, of Harvard, is meeting with much favor. There is so much detail in each medical specialty that no one student can master the subjects sufficiently to enter into a general practice of all. Special teaching is necessary even after the graduate has finished his hospital training.

A full and thorough course of two or three years should be required of all students in the major branch of medicine, and a satisfactory examination should be required, after which the student should select the specialty he desires to practice and with the aid of the Dean select such other branches as will fit in with those of his specialty. His special training in the stomatologic hospital (dental clinic) can be continued in the schools, which are now so well equipped for special work. The courses in both general and special medicine should continue throughout the year, divided into four quarters. By this method, the student can better select the course best adapted to each year's curriculum.

### SPECIAL DEGREES FOR SPECIAL WORK.

Should the elective system eventually be adopted, then all the "ologies" would specialize after the second or third year of medical study. This would simplify matters and the stomatologist would then stand on an equal footing with other specialists. It would not be a bad plan also to confer the special degree of D.D.S. on such students as become proficient in special work. The specialties should be pursued as they are at the present time. Having finished his medical course, his special teaching should be shortened one year at lesat. The details of special instruction can only be worked out in the dental hospital on those students who have acquired a medical course. The student's special instruction should continue until he has become proficient in the different departments and has come up to a given standard. In other words, merit should be recognized, whether it be six months or three years. The standard of requirement should be established by a committee consisting of one delegate from each university stomatological department, or still better, by the state board of dental examiners.

### REGISTRATION OF SPECIALISTS.

The state board of medical examiners should register the graduate of medicine and the particular specialty he has become proficient in. The law should require him to practice that specialty. Should he find later that he is better adapted to practice some other specialty, he should pursue studies along that line, stand another examination before the state board of medical examiners and be licensed to practice that specialty.

It is to be hoped that the Council on Medical Education and the Association of American Medical Colleges will soon consider the return of the prodigal son to the mother profession, and not only make provision for his welcome back into the hous hold, but also provide for the other brothers (specialists) that the fatted calf may be distributed equally to all children so that they may all eat and drink of the knowledge of medicine and make merry.—*Journal A. M. A.*, June 25, 1910.

